# Recent Progress of Ferroptosis in Lung Diseases

**DOI:** 10.3389/fcell.2021.789517

**Published:** 2021-11-16

**Authors:** Shangjiang Yu, Jinqiu Jia, Jinyu Zheng, Yiyang Zhou, Danyun Jia, Junlu Wang

**Affiliations:** ^1^ Department of Clinical Medicine, Wenzhou Medical University, The First Affiliated Hospital of Wenzhou Medical University, Wenzhou, China; ^2^ Department of Pediatrics, Taizhou Women and Children’s Hospital of Wenzhou Medical University, Taizhou, China; ^3^ Department of Anesthesiology, The First Affiliated Hospital of Wenzhou Medical University, Wenzhou, China

**Keywords:** ferroptosis, mechanism, lung diseases, iron-dependent, programmed cell death

## Abstract

Ferroptosis is a new form of programmed cell death due to iron-dependent excess accumulation of lipid peroxides and differs from other programmed cell deaths in morphological and biochemical characteristics. The process of ferroptosis is precisely regulated by iron metabolism, lipid metabolism, amino acid metabolism, and numerous signaling pathways, and plays a complex role in many pathophysiological processes. Recent studies have found that ferroptosis is closely associated with the development and progression of many lung diseases, including acute lung injury, pulmonary ischemia-reperfusion injury, lung cancer, chronic obstructive pulmonary disease, and pulmonary fibrosis. Here, we present a review of the main regulatory mechanisms of ferroptosis and its research progress in the pathogenesis and treatment of lung diseases, with the aim of providing new ideas for basic and clinical research of lung-related diseases.

## Introduction

Ferroptosis is a new type of iron-dependent programmed cell death caused by lipid peroxidation (LPO), which differs from other programmed cell deaths in morphology and biochemical characteristics. In terms of morphology, there is no chromatin agglutination and nuclear fragmentation as opposed to the process of cell apoptosis, nor the formation of autophagic vacuoles with two-layered membrane structures seen during autophagy, or swelling of organelles and rupture of the plasma membrane during necrosis. Its main morphological characteristics are mitochondrial shrinkage and increased mitochondrial membrane density, accompanied by the reduction or disappearance of mitochondrial cristae and outer membrane disintegration (Dixon et al., 2012). The corresponding biochemical manifestations are the accumulation of reactive oxygen species (ROS) and iron ions, the decrease of cysteine uptake and glutathione (GSH) synthesis, activation of mitogen-activated protein kinase system and release of arachidonic acid. Ferroptosis is associated with a variety of lung diseases and is regulated by polygenic pathways. This paper will review the research progress of ferroptosis in lung diseases and provide a new perspective for the study of pathogenesis and clinical treatment of lung diseases.

## The Mechanism of Ferroptosis (Figure 1)

### Cystine/Glutamate Reverse Transport System and Glutathione Peroxidase 4 Pathway

**FIGURE 1:**
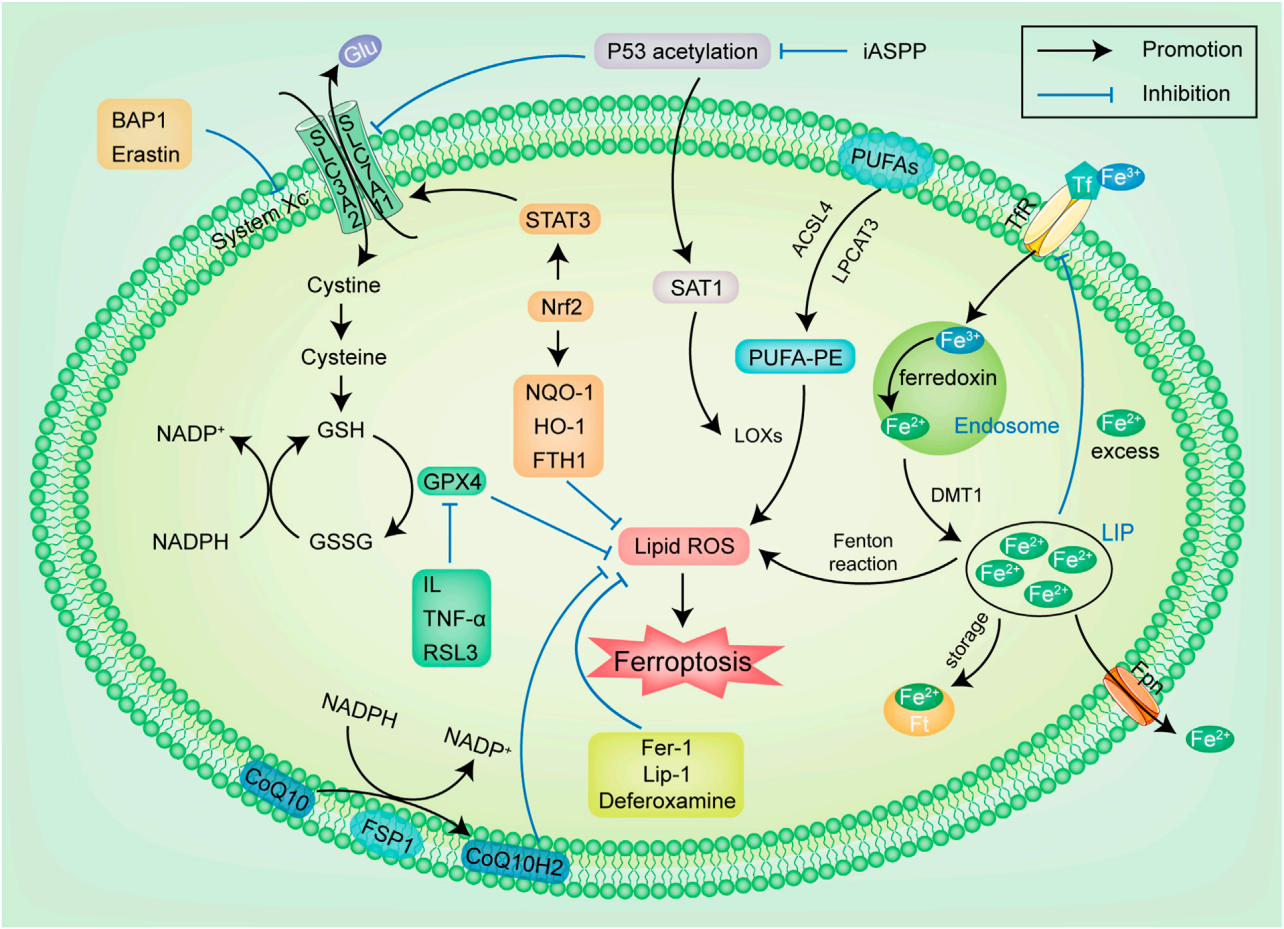
The regulatory pathways of ferroptosis. The figure shows the regulation pathway of ferroptosis, which can be simply summarized into two types. The first type is the metabolic pathways related to ferroptosis, such as iron metabolism, lipid metabolism and amino acid metabolism pathways; The second type is the signaling pathway related to ferroptosis, such as p53, Nrf2, FSP1 pathways. Glu: glutamic acid; GSH: glutathione; GSSG: oxidized glutathione; NADPH: nicotinamide adenine dinucleotide phosphate; GPX4: glutathione peroxidase 4; IL: Interleukin; TNF-α: tumor necrosis factor-α; RSL3: RAS-selective lethal 3; Nrf2: nuclear factor erythroid-derived 2; STAT3: signal transducer and activator of transcription-3; NQO-1: NADPH-quinone oxidoreductase-1; iASPP: inhibitor of apoptosis-stimulating protein of p53; SAT1: spermidine/spermine N1-acetyltransferase 1; LOXs: lipoxygenases; ROS: reactive oxygen species; FTH1: ferritin heavy chain 1; DMT1: divalent metal ion transporter-1 CoQ10: coenzyme Q10; Fpn: ferroportin; TfR: transferrin receptor; Ft: ferritin; LIP: labile iron pool; Lip-1: liproxstatin-1; Fer-1: ferrostatin-1; FSP1: ferroptosis suppressor protein 1.

Figure 1 The cystine/glutamate reverse transporter (system Xc-) exists on the cell membrane and is composed of SLC3A2 and SLC7A11 (Dixon et al., 2014), which are linked by a disulfide bond. System Xc-transports cystine inside and glutamic acid (Glu) outside in a ratio of 1:1. Cystine is reduced to cysteine after entering the cell, and cysteine is used as one of the raw materials to synthesize GSH. GSH is a necessary substrate for glutathione peroxidase 4 (GPX4) to degrade LPO (Wan et al., 2007). GPX4 is the only known anti membrane peroxidase that can reduce lipoxygenases (LOXs) and prevent excessive activation. It can also remove LPO produced by iron accumulation and effectively inhibit cell membrane damage caused by LPO (Stoyanovsky et al., 2019). In this channel, these following links can be the targets of regulating ferroptosis: ① System Xc-: The ferroptosis inducer Erastin targets system Xc-. Erastin reduces the level of antioxidant enzymes by inhibiting cystine uptake by system Xc-, thus inducing ferroptosis (Chen et al., 2015). The tumor suppressor BRCA1-associated protein 1 inhibits the growth of cancer cells by regulating the expression of SLC7A11 (Zhang et al., 2019), and its effect is similar to Erastin. It can inhibit the expression of SLC7A11, reduce the cystine uptake and the synthesis of GSH, leading to the accumulation of LPO, and increase the sensitivity of cancer cells to ferroptosis. A study has found that glutamine (Gln) has a significant impact on the occurrence of ferroptosis: Gln generates Glu under the action of glutaminase. When there is a high concentration of Glu outside the cell, the effect of system Xc-is inhibited, thus promoting ferroptosis (Murphy et al., 1989). α-Ketoglutarate is the product of Glu under the action of glutamate dehydrogenase, which can also effectively induce ferroptosis. Gln inhibitor can inhibit ferroptosis induced by protein kinase. ② GSH: As a reducing agent of peroxide and disulfide, GSH maintains the homeostasis of oxidation and reduction in organisms (Deponte, 2013). Nicotinamide adenine dinucleotide phosphate (NADPH) inhibits ferroptosis by reducing oxidized glutathione disulfide to GSH and maintaining the reduced state of GSH (Shimada et al., 2016). NADPH is also considered one of the sensitive markers of ferroptosis, and its depletion is considered a potential cause of ferroptosis. ③ GPX4: GPX4 knockout cells are more prone to ferroptosis induced by ROS. GPX4 inhibitor RSL3, as a ferroptosis inducer of head and neck cancer (HNC) cells, can be bind to GPX4 protein and degrade it, thus increasing LPO production and inducing ferroptosis (Dächert et al., 2016).

### Lipid Metabolism Pathway

LPO is the key link in triggering ferroptosis. Excessive accumulation of LPO will cause plasma membrane damage and eventually lead to cell ferroptosis. Polyunsaturated fatty acids (PUFAs) are more prone to oxidation to produce peroxy groups due to their labile double bonds, and are also one of the key mechanisms of ferroptosis. When PUFAs are present in large amounts, more lipid peroxides are produced, aggravating the degree of ferroptosis (D’Herde et al., 2017). With the help of two enzymes, acyl-CoA synthetase long-chain family member 4 (ACSL4) and lysophosphatidylcholine acyltransferase 3 (LPCAT3) (Dixon et al., 2015), PUFAs in cell membranes undergo synthesis, esterification, and incorporation into membrane phospholipids to generate PUFA-PEs. PUFA-PEs can form LPO by promoting LOXs mediated enzymatic reaction, and LOXs can also promote the peroxidation of PUFAs (Yang et al., 2016), triggering ferroptosis.

### Iron Metabolism Pathway

Regulation of endogenous iron homeostasis is achieved through the iron regulatory protein (IRP) system. The IRP system can sense the concentration of free Fe^2+^ in cells, which is composed of transferrin receptor (TfR), divalent metal ion transporter-1 (DMT1), transferrin, ferroportin1 and ferritin (Ft), etc (Andrews and Schmidt, 2007). Fe^3+^ in the peripheral circulation binds to the TfR on the cell membrane, enters the endosomes, is reduced to Fe^2+^ by ferredoxin, and then disintegrated from the endosomes mediated by DMT1 and released into the cytoplasm for storage in the labile iron pool (LIP) (Gao et al., 2015). LIP can inhibit the expression of TfR1 through negative feedback, reducing the incorporation of Fe^3+^(Brissot et al., 2012). Excessive Fe^2+^ in LIP and hydrogen peroxide produce hydroxyl radicals and ROS through Fenton reaction and iron-dependent oxidase action, which is the initiation of ferroptosis (Xie et al., 2016). Not only does the electron-receiving ROS interact with lipids to produce LPO (Dächert et al., 2016), but it also aggravates the mutational damage of mitochondria (Al-Qenaei et al., 2014). Cell membrane and plasma membrane are more sensitive to LPO, and the presence of Fe^2+^ will aggravate cellular oxidative stress, promoting the ferroptosis process. Antioxidants and deferoxamine can significantly inhibit the ferroptosis induced by Erastin, indicating that iron plays an important role in the occurrence and progress of ferroptosis (Dixon et al., 2012).

### P53 Gene

P53 is a human tumor suppressor gene, which plays an important role in cell growth, apoptosis and DNA repair. P53 can prevent the replication of damaged DNA, block the cell cycle, and allow DNA repair-related enzymes to intervene. If repair is not completed, p53 induces apoptosis of damaged cells, thereby controlling cell proliferation and preventing the occurrence of cancer cells. The expression level of p53 is closely related to the occurrence of ferroptosis. In lung cancer, after p53 is acetylated, the key to p53 mediated ferroptosis and tumor suppression lies in its direct inhibitory effect on System Xc- (Jiang et al., 2015). P53 can regulate ferroptosis in the following links:①SLC7A11: After p53 acetylation, it can significantly reduce the expression of SLC7A11, inhibiting System Xc-activity, and blocking the uptake of cystine in the upstream pathway to induce ferroptosis (Wang et al., 2016). ②glutaminase 2 (GLs2): The Gls2 gene contains a functional p53 DNA binding element in its promoter region. p53 can increase the activity of Gls2, enhance mitochondrial respiration and ATP production, catalyze mass production of Glu, inhibit System Xc-activity (Tang et al., 2021), and induce ferroptosis by blocking the uptake of cystine in the upstream pathway. ③SAT1: spermidine/spermine N1-acetyltransferase 1 (SAT1) is the transcription target of p53 (Ou et al., 2016). In the case of ROS-induced oxidation stress, p53 mediated activation of SAT1 can lead to ferroptosis. By enhancing the expression of SAT1, p53 promotes ferroptosis and acts as a cancer suppressor.

### Ferroptosis Suppressor Protein 1

Ferroptosis suppressor protein 1 (FSP1) is located in the cytoplasmic membrane and exerts an anti-ferroptosis effect through its N-terminal myristoylated motif. It is the redox of NADPH-dependent coenzyme Q (CoQ) oxidoreductase. FSP1 maintains the reduced form of CoQ at the plasma membrane, which antioxidant activity is not dependent on GSH. The reduced form of CoQ traps lipid peroxyl radicals and prevents the diffusion of LPO (Doll et al., 2019). In GPX4 knockout cells, the presence of FSP1 can significantly reduce LPO production. Overexpression of FSP1 inhibits LPO and ferroptosis. Therefore, the level of FSP1 can be used as a biological indicator to measure the ability of various cancer cells to resist ferroptosis (Bersuker et al., 2019).

### Nuclear Transcription Factor-2

Nuclear transcription factor-2 (Nrf2) is a critical regulator of antioxidant response (Ma, 2013). Under normal conditions, Nrf2 is low-level. Once oxidative stress occurs, Nrf2 quickly moves from the cytoplasm to the nucleus and combines with antioxidant response elements/electrophilic response elements to activate antioxidant genes and downstream enzymes to exert antioxidant effects, thereby inhibiting ferroptosis (Pi et al., 2008; Suzuki et al., 2013). It has been proved that the Keap1-Nrf2 pathway up-regulates multiple genes: NADPH-quinone oxidoreductase-1, heme oxygenase 1, and Ft, etc., which play an important role in protecting liver cancer cells against ferroptosis (Sun et al., 2016). After artesunate treatment, the level of GSH in HNC cells was significantly reduced, increasing ROS level in cells and inducing ferroptosis. Activation of Nrf2 can effectively enhance the resistance of HNC cells to artesunate (Roh et al., 2017). In cisplatin-resistant HNC cells, the level of basic Nrf2 was detected to be higher, and inhibition of Nrf2 also reversed the resistance of HNC cells to ferroptosis induced by GPX4 inhibitors (Shin et al., 2018).

### Mediated by Inflammation

Various types of cell death have been implicated in the regulation of inflammation. When ferroptosis occurs, it can mediate inflammation immunogenically, intervening in the immune system to dispose of dying cells. Intracellular components undergoing ferroptosis can release ATP, nucleotides, and pro-inflammatory cytokines from pro-inflammatory damage-associated molecular patterns (DAMPs) to promote the development of necroinflammation (Linkermann et al., 2014). In addition, the oxidative stress response that occurs during ferroptosis can trigger the large expression of cytokines such as Nrf2, NF-κB, tumor necrosis factor-α(TNF-α), etc. (Reuter et al., 2010), leading to inflammation and immune cell chemotaxis. GSH and GPX4 are key regulators of ferroptosis and are also critical in mediating inflammatory responses. Decreased GPX4 expression in ferroptosis leads to increased expression of 12-LOX and cyclooxygenase, which in turn triggers an inflammatory response (Chen et al., 2003). Up-regulation of GPX4 can reduce the oxidation of arachidonic acid and inhibit the NF-κB pathway to inhibit ferroptosis and inflammation (Li et al., 2018a). GSH is necessary to suppress ROS and combat inflammatory damage, activated T cells need GSH to metabolize the ROS they produce to prevent cell damage, but it appears to decline dramatically in ferroptosis (Mak et al., 2017). GPX4-deficient T cells rapidly accumulated membrane LPO, underwent cell death driven by ferroptosis and subsequently affect immune function (Matsushita et al., 2015). High levels of ROS generated by iron overload can polarize macrophages to a pro-inflammatory phenotype by promoting tumor suppressor p53 acetylation (Zhou et al., 2018). These results indicate that ferroptosis may be the initiating factor of inflammation, or at least has a pro-inflammatory effect. However, whether necroinflammation occurs in the ferroptosis process itself or in response to cell rupture and DAMP release remains to be studied. Other studies have shown that some inflammatory cytokines such as TNF, PGE, Interleukin-1(IL-1), IL-6, etc., can directly affect the level and activity of GPX4 in cells (Kim et al., 2008). A significant decrease in GPX4 expression was observed after TNF treatment of the cells, while potentially triggering ferroptosis (Wen et al., 2019). The pro-inflammatory metabolite leukotrienes derived from LOXs can also indirectly promote ferroptosis (Proneth and Conrad, 2019). These illustrate how ferroptosis and inflammation may be complementary. At present, the research on necrotizing inflammation in ferroptosis is still in the early stage and needs further exploration.

## Ferroptosis and Lung-Related Diseases (Table 1)

### Ferroptosis and Acute Lung Injury

Table 1 The lung is susceptible to oxidative stress, so there are high concentrations of antioxidant GSH and ascorbic acid in cells. In the occurrence of acute respiratory distress syndrome (ARDS), it is found that protein and lipid damage of lung epithelial cells, alveolar edema with a large number of leukocyte infiltrations caused by ROS, and signs of ferroptosis with reduced ascorbic acid and GSH can be observed (Chabot et al., 1998). In the mouse models of acute lung injury (ALI) induced by oleic acid and mouse models of ALI induced by intestinal ischemia-reperfusion (IR), the phenomena of mitochondrial shrinkage and mitochondrial membrane rupture were observed in type II alveolar epithelial cells. Feature indicators of ferroptosis, such as iron overload, GSH consumption and malondialdehyde (MDA) accumulation, and down-regulation of GPX4 and Ft protein expression levels in lung tissue were also detected (Zhou et al., 2019; Dong et al., 2020). In the mouse model of ALI induced by intestinal IR, both inhibitor of apoptosis-stimulating protein of p53 (iASPP) and Nrf2 exerted therapeutic effects. iASPP inhibits ferroptosis and alleviates ALI, while iASPP-mediated protection relies on Nrf2 signal transduction (Li et al., 2020). The increased expression of Nrf2 in the ALI model alleviates the decrease in GPX4 levels and promotes the phosphorylation of signal transducer and activator of transcription-3 (STAT3). STAT3 enhances the antioxidant capacity of cells by activating SLC7A11, thereby alleviating the pathological process related to ALI (Qiang et al., 2020). In ALI models induced by Lipopolysaccharide (LPS) cells, the contents of MDA, 4-HNE and total iron were significantly increased, and the expressions of SLC7A11 and GPX4 were decreased. Ferroptosis inhibitor ferrostatin-1 has a reduced effect on LPS-induced ALI, indicating that ferroptosis is involved in LPS-induced ALI (Liu et al., 2020a). When Panaxadiol (PX) extracted from ginseng root was used to treat LPS-induced ALI, the results showed that PX effectively alleviated the pathological changes of ALI in mice, and PX inhibited ferroptosis had alleviated the symptoms of ALI by up-regulating keap1-Nrf2/HO-1 pathway (Li et al., 2021a). These studies preliminarily confirmed the potential effect of inhibiting ferroptosis in the treatment of ALI, and provided new ideas for clinical treatment of ALI. Neutrophil-dominated leukocyte infiltration in the alveoli of ALI patients should also not be ignored. Targeting induced ferroptosis in neutrophils is important to improve respiratory function, control the further progression of ALI, and reduce case fatality.

**TABLE 1:** Key mechanisms and regulators of ferroptosis in lung diseases.

Disease	Key Mechanisms	Regulator	References
ALI	Iron overload	Inhibitor: iASPP Fer-1 PX	Zhou et al. (2019); Liu et al. (2020a); Dong et al. (2020); Li et al. (2020); Li et al. (2021a)
	Decrease GSH and GPX4 protein levels		
	Decrease SLC7A11 expression		
	Inflammation		
LIRI	Accumulation of lipid ROS	Inhibitor: rosiglitazone	Li et al. (2018b); Xu et al. (2020)
	Inflammation		
COPD	Iron overload	Inhibitor: Fer-1	Ghio et al. (2008); Wang and Tang (2019); Yoshida et al. (2019)
	Decrease GSH and GPX4 protein levels	Deferoxamine	
PF	Decrease GPX4 protein level	Inhibitor	Kim et al., (2010); Gong et al. (2019)
	Increase ROS level	Fer-1	
	Iron overload	Lip-1	
Lung cancer	Upregulate the expression of System Xc- and FSP1 level Decrease p53 and reduce intracellular Fe^2+^ and ROS level	Inducer	Huang et al. (2005); Mao et al. (2018); Bersuker et al. (2019); Wang et al. (2019); Yokoi et al. (2019); Chen et al. (2020)
		Erianin	
		Cisplatin	
		Erastin	
Asthma	Increase 15-LOX1 expression Inflammation	Inducer	Zhao et al. (2011); Wu et al. (2020)
		Erastin	
		RSL3	
PT	Decrease GSH and GPX4 protein levels	Inhibitor	Seyedrezazadeh et al. (2008); Amaral et al. (2019); Pan et al. (2020)
	Increase free iron and LPO	Fer-1	
	Inflammation	Vitamin E	
COVID-19	Increase TfR content	Inhibitor	Zhao et al. (2020b); Cavezzi et al. (2020); Jaiswal et al., (2020); Silvagno et al. (2020)
	Iron overload	Deferoxamine	
	Decrease GSH and GPX4 protein levels	N-acetylcysteine	

ALI: acute lung injury; GSH: glutathione; GPX4: glutathione peroxidase 4; iASPP: inhibitor of apoptosis-stimulating protein of p53; Fer-1: ferrostatin-1; PX: Panaxadiol; Nrf2: nuclear factor erythroid-derived 2; LIRI: lung ischemia-reperfusion injury; ROS: reactive oxygen species; COPD: chronic obstructive pulmonary disease; Lip-1: liproxstatin-1; TfR: transferrin receptor; PF: pulmonary fibrosis; system Xc-: cystine/glutamate reverse transporter; FSP1: ferroptosis suppressor protein 1; RSL3: RAS-selective lethal 3; 15-LOX1: 15-lipoxygenase1; PT: Pulmonary tuberculosis; COVID-19: Corona Virus Disease 2019

### Ferroptosis and Lung Ischemia-Reperfusion Injury

The *in vitro* model of IR generates a large amount of ROS, which may recruit pro-inflammatory leukocytes and destroy the membrane integrity of plasma endothelial and epithelial cells (Li et al., 2018b), resulting in alveolar injury and barrier function damage. In the IR group model of experimental rats, ferroptosis signs such as mitochondrial oxidative stress and morphological damage were present. Human bovine pyridine apelin-13, which is used to regulate mitochondrial function in myocardial and brain IR damage, can effectively reduce the production of inflammatory factors and reduce pulmonary edema by reducing ROS and increasing the expression level of uncoupling protein-2 on mitochondrial intima (Xia et al., 2021). The symptoms of ferroptosis were alleviated by the use of an ACSL4 inhibitor. Knockdown of ACSL4 can reduce the accumulation of lipid ROS and reduce the sensitivity of lung epithelial cells to ferroptosis (Xu et al., 2020). Due to the important role of oxidative stress in IR injury, in lung transplantation, lung resection and other operations that may cause LIRI, the cellular damage caused by ferroptosis can be avoided by attenuating the oxidative stress reaction, thereby reducing the risk of patients during the operation. Targeting the inhibition of ferroptosis might be a new avenue for anti-IR injury treatment.

### Ferroptosis and Chronic Obstructive Pulmonary Disease

Chronic obstructive pulmonary disease (COPD) is a disease with airflow obstruction as the main pathological change, which can cause irreversible lung injury. The etiology of COPD is not clear, and it is currently believed to be related to the abnormal inflammatory response of harmful gases and harmful particles. Cigarette smoke (CS) has an important effect on iron homeostasis in the lung. Exposure of mouse and human bronchial epithelial cells to CS increased the concentrations of iron, Ft, serum ferritin and non-heme iron in lung cells (Ghio et al., 2008). Endoplasmic reticulum stress and mitochondrial dysfunction were found in the cytoplasm (Park et al., 2019), then ferroptosis occurred in bronchial epithelial cells. After being induced by CS, the autophagy of Ft mediated by nuclear receptor coactivator 4 (NCOA4) leads to the accumulation of free iron. The reduction of the activity level of GPX4 and insufficient GSH, etc., all play an important role in the pathogenesis of COPD, leading to HBEC peroxidation and ferroptosis (Dowdle et al., 2014; Yoshida et al., 2019). Experimental interventions using ferroptosis-associated inhibitors such as deferoxamine and ferrostatin-1 have shown some efficacy. PM2.5 is also one of the potential risk factors for COPD. After inhaling PM2.5 particles, the content of iron and the concentration of ROS in human endothelial cells increase, while the levels of GSH and NADPH decrease. Changes in the expression of TfR and Ft are the main causes of iron homeostasis imbalance, which in turn lead to ferroptosis (Wang and Tang, 2019). Ferrostatin-1 and iron chelator deferoxamine can improve the reduction of GSH and NADPH levels in endothelial cells. The above studies have confirmed that ferroptosis is an important damage mechanism of COPD, and finding a highly specific ferroptosis inhibitor to control the occurrence and development of COPD is the key to future research.

### Ferroptosis and Pulmonary Fibrosis

Pulmonary fibrosis (PF) is an interstitial lung disease that develops from long-term inhalation of dust, the use of drugs such as amiodarone, bleomycin, or lung injury caused by radiation therapy. The formation of fibrotic foci is a prominent pathological feature of PF (Richeldi et al., 2017). Collagen deposition appeared in lung tissue exposed to radiation, and signs of ferroptosis with decreased GPX4 expression and increased ROS were observed (Li et al., 2019). Erastin promotes the differentiation of fibroblasts to myofibroblasts by increasing LPO, ROS levels and inhibiting GPX4 expression (Gong et al., 2019), which in turn induces collagen accumulation and destruction of alveolar structure to induce PF. Normal lung macrophages have a strong antioxidant capacity. Their intralysosomal Ft enhances the stability of lysosomes via iron chelation, blocks the Fenton reaction, and alleviates oxidant-induced lysosomal damage and cell death (Persson et al., 2001; Persson et al., 2011). Furthermore, there is an oxidative/antioxidant imbalance in PF patients. Pulmonary macrophage hemosiderin and MDA accumulation were significantly increased in PF patients affected by CS exposure. Iron deposition has been observed in lung histological sections and is significantly associated with advanced pulmonary hypertension in clinical stages of IPF patients (Kim et al., 2010). Thus iron homeostasis may play a key role in epithelial cell damage and fibroblast proliferation induced by oxidative stress (Rahman et al., 1999). Therefore, in future clinical practice, one can try to delay the progression of fibrosis by regulating iron metabolism and controlling lung iron homeostasis in PF patients.

### Ferroptosis and Lung Cancer

Lung cancer is the cancer with the highest incidence and the highest mortality in China. Recent studies have found that lung cancer is closely related to ferroptosis, as lung cancer cells are in a state of ferroptosis-inhibition. Lung cancer cells need stronger anti-oxidation and anti ferroptosis ability to survive in the environment of high oxidation for a long time. ① By up-regulating system Xc-, lung cancer cells can improve the antioxidant effect of the downstream pathway, increase the antioxidant capacity of cells, and inhibit the occurrence of ferroptosis (Huang et al., 2005). ② FSP1 is highly expressed in lung cancer cells, inhibiting ferroptosis and allowing lung cancer cells to grow (Bersuker et al., 2019). ③ Many genomic mutation regions in cancer cells are transcribed into long non-coding RNA (lncRNA). The expression of lncRNA affects cell homeostasis and is related to different types of cancer (Huarte, 2015). Among them, lncRNA LINC00336 is up-regulated in lung cancer. Overexpression of lncRNA LINC00336 significantly reduces the concentration of intracellular Fe^2+^, ROS and mitochondrial superoxide, which inhibits ferroptosis by acting as a competitive endogenous RNA. It is also observed that overexpression of lncRNA LINC00336 limits ferroptosis induced by GPX4-inhibitor RSL3 in lung adenocarcinoma cells (Wang et al., 2019; Jiang et al., 2021). In addition, the down-regulation of lncRNA P53RRA in cancer removes p53 and weakens the ferroptosis in lung adenocarcinoma and lung squamous cell carcinoma, which is induced by p53 (Mao et al., 2018). ④ The overexpression of thioredoxin-2 (TXN2) and the depletion of haptoglobin (HP) can make lung cancer cells more resistant to ferroptosis induced by Erastin or RSL3 (Li et al., 2021b). Erianin, an effective component extracted from Dendrobium chrysotoxum Lindl, can activate calmodulin (CaM) in lung cancer cells. CaM can regulate L-type voltage-dependent Ca^2+^ channels. Increased Ca^2+^ uptake leads to increased levels of ROS and Fe^2+^(Kiefmann et al., 2017). By causing excessive accumulation of ROS and depletion of GSH in lung cancer cells, it induces ferroptosis of lung cancer cells and inhibits lung cancer cell migration (Chen et al., 2020). Cisplatin is currently widely used in the treatment of lung cancer, with ferroptosis as the target. Cisplatin is an inducer of ferroptosis and apoptosis in lung adenocarcinoma cells, and its mechanism is to cause reduced GSH consumption and GPX4 inactivation. In addition, the combination of cisplatin and Erastin has a significant synergistic effect on anti-tumor activity, which may be related to the down-regulation of GSH and the inactivation of GPX to stimulate the Erastin pathway (Yokoi et al., 2019). The above experiments have verified that lung cancer cells enhance their resistance to ferroptosis through multiple genetic pathways. The follow-up research can focus on more effective and targeted intervention in such regulatory pathways, making lung cancer cells sensitive to ferroptosis, inhibiting its growth and prolonging the survival of lung cancer patients.

### Ferroptosis and Asthma

Asthma is a disease characterized by airway inflammation and airway hyperreactivity, in which oxidative stress plays an important role. Upon activation of T cells by antigen-presenting cells, Th2 secrete IL. B lymphocytes are activated to synthesize specific IgE and produce a variety of active mediators in the process of type I hypersensitivity, leading to airway smooth muscle contraction, mucus secretion, and inflammatory cell infiltration, thereby causing asthma symptoms. On the other hand, IL directly activates mast cells, eosinophils (Eos), macrophages to accumulate in the airways and release inflammatory factors leading to chronic airway inflammation. In a highly oxidized microenvironment, H2O2 can up-regulate Th2-driven airway inflammation by influencing the secretion of Th1 and Th2 cytokines and enhancing the airway’s hyperreactivity, which makes the above symptoms prolonged and aggravated (Frossi et al., 2008). Airway inflammation and the hyperoxidative state of asthma patients are also crucial factors contributing to ferroptosis. The fresh bristle hematopoietic stem cells and airway epithelial cells of asthma patients showed high expression of 15-LOX1. The combination of 15-LOX1 and Phosphatidylethanolamine binding protein 1 (PEBP1) can activate extracellular regulatory protein kinases, inducing autophagy and ferroptosis (Zhao et al., 2011; Zhao et al., 2020a). In tissues where ferroptosis occurred, macrophages were found to be significantly activated and released pro-inflammatory substances. Generally speaking, asthma exacerbations had a significant association with iron mortality. Recent understanding of asthma pointed out that Eos not only play the role of effector cells, but also have immunomodulatory effects. The average survival cycle of Eos is approximately 2–5 days, while Eos survival is prolonged in the inflammatory microenvironment of asthma. Ferroptosis-inducing agents induce Eos ferroptosis *via* an atypical pathway and are synergistic with the glucocorticoids in triggering Eos death. Treatment targeting Eos ferroptosis appears to be effective in controlling allergic airway inflammation such as asthma and may reduce the dose and adverse effects of glucocorticoids (Wu et al., 2020). In hyperneutrophilic asthma patients resistant to corticosteroid hormones, targeted induction of ferroptosis in neutrophils would be more clinically valuable. In conclusion, the idea of inducing ferroptosis of inflammatory cells to alleviate airway inflammation and reduce the amount of hormones has great potential in future studies of asthma.

### Ferroptosis and Pulmonary Tuberculosis

Pulmonary tuberculosis is still a major infectious disease that seriously endangers human health, and it is now showing a trend of deterioration in the world. After the human body inhales *Mycobacterium tuberculosis* (Mtb), the macrophages in the alveoli are the first to secrete large amounts of IL-1, IL-6, and TNF-α, allowing lymphocytes and monocytes to aggregate, forming combined granulomas to limit the spread of Mtb. Th1 cells play an immune protective role and promote the function of macrophages. The contest between Mtb and macrophages determines the direction of tuberculosis. Studies have found that Mtb-induced macrophage death was associated with decreased GSH and GPX4 levels and increased free iron, mitochondrial superoxide, and LPO. The destruction of macrophages by Mtb was reduced after Ferrostatin-1 treatment. Similar manifestations were also seen in necrosis of the lung, demonstrating that ferroptosis is the primary mechanism of necrosis in Mtb infection (Amaral et al., 2019). Ferrostatin-1 has a certain antibacterial effect. It reduced lung necrosis in infected mice and significantly reduced the number of bacteria in the lungs. At present, the conventional treatment of tuberculosis requires prolonged use of antibiotics, with the risk of recurrent infections and the emergence of drug-resistant strains. The main therapeutic drugs, isoniazid and rifampicin, both consume a large amount of GSH during liver metabolism, causing LPO and death of hepatocytes (Pan et al., 2020). In view of the above-mentioned treatment problems, we urgently need to find a faster and safer treatment. Vitamin E as an antioxidant can reduce non-heme iron from Fe^3+^ state to Fe^2+^ state to inhibit 15-LOX1 (Hinman et al., 2018), thereby inhibiting the ferroptosis pathway mediated by 15-LOX1 (Kagan et al., 2017). In clinical trials, vitamin E was used to intervene in tuberculosis patients for 2 months. It was found that oxidative stress was reduced, and the overall antioxidant status of tuberculosis patients was improved (Seyedrezazadeh et al., 2008). *Pseudomonas aeruginosa*, which is also an infectious lung pathogen, can express LOXs after infection, causing ferroptosis of human bronchial epithelial cells (Dar et al., 2018). So the inhibition of LOXs-driven ferroptotic pathways might be a potential target for treating pulmonary infections such as tuberculosis and *Pseudomonas aeruginosa*.

### Ferroptosis and Corona Virus Disease 2019

Corona Virus Disease 2019 (COVID-19) is an infectious inflammatory disease. In the early stage of infection, the symptoms are mostly fever, cough, muscle aches, and fatigue (Huang et al., 2020a). Severe cases include dangerous conditions such as ARDS, septic shock, severe metabolic acidosis, and hypercoagulability. Respiratory failure is the most common cause of death in severe patients with COVID-19.

The pathological changes of COVID-19 mainly include hemoglobin damage, hypoxia, methemoglobinemia, and cellular iron overload (Cavezzi et al., 2020; Colafrancesco et al., 2020). Iron is considered to be a key factor in the pathogenesis of COVID-19, and elevated serum Ft is closely related to poor prognosis (Huang et al., 2020b; Edeas et al., 2020; Zhou et al., 2020). In a number of clinical studies of covid-19 patients, almost all patients had decreased serum iron level, which was speculated to be due to the high expression of TfR (Zhao et al., 2020b). TfR can transport serum iron into cells to accumulate. The expression level of TfR is related to age, and the expression level of TfR in males is significantly higher than that in females, which may explain the higher infection rate and mortality of male elderly patients among COVID-19 patients (Frazer and Anderson, 2014; Borges do Nascimento et al., 2020; McLaughlin et al., 2020). By observing the dependence of Severe Acute Respiratory Syndrome Corona Virus 2 (SARS-CoV-2) replication on iron and the regulation of virus on host iron metabolism, it was speculated that cell iron was the key to the invasiveness and survivability of the virus. Then, it was proved that iron was involved in several key steps of SARS-CoV-2 replication (Liu et al., 2020b).

Iron chelator has immunomodulatory and anti-inflammatory effects, and can bind to several receptors used by SARS-CoV-2 to prevent it from entering host cells. The therapeutic mechanism of iron chelators also includes down-regulation of hepcidin, binding of free iron, or depletion of iron from Ft, thus interfering with virus replication (Cavezzi et al., 2020). After SARS-CoV-2 infects cells, it is found that the expression levels of GSH and GPX4 are significantly reduced. The inflammatory response caused by SARS-CoV-2 is related to the cell damage produced by ROS, suggesting the correlation between COVID-19 and ferroptosis (Silvagno et al., 2020). The application of reducing agents such as methemoglobin reductase, ascorbic acid and GSH can reduce the trivalent iron in hemoglobin to ferrous iron, so as to restore the ability of hemoglobin to combine with oxygen and relieve the symptoms of hypoxia (Muhoberac, 2020). N-acetylcysteine has the effect of scavenging ROS and is an effective precursor of GSH, which can be considered an auxiliary drug to reduce oxidative stress in patients with COVID-19 (Jaiswal et al., 2020).

Reducing the concentration of intracellular iron and increasing the level of reducing agents is the most fundamental treatment to alleviate the redox and ROS damage of cells, especially in severe COVID-19 cases like ARDS. The correct selection of chelating agents and reducing agents to prevent the early formation of ROS is an important control measure.

## Conclusion and Outlook

Ferroptosis is a new way of programmed cell death caused by the excessive accumulation of iron-dependent LPO, involving various metabolic pathways such as amino acid metabolism, lipid metabolism, and iron metabolism. With the progress of ferroptosis research, more and more influencing pathways and regulatory factors have emerged, forming a complex network of ferroptosis occurrence and development. A large number of experimental studies have revealed that ferroptosis is involved in the morphological changes and pathological processes of various diseases. In ALI, IR, COPD, and pulmonary infectious diseases where ferroptosis is clearly involved in the injury mechanism, intervention at a certain point in the ferroptosis network may play a role in the early prevention of the disease. This would lead to the improvement of clinical symptoms and control of the development of the disease course. In the future, we can also try to explore whether the combined use of ferroptosis inhibitors in various ways will achieve better therapeutic effects. Therefore, in the context of the many achievements in animal model experiments, it is essential to perform more in-depth studies on the mechanisms and targets of action of ferroptosis inhibitors. More efficient and specific modulation of cellular ferroptosis is key for future research.

## References

[B1] Al-Qenaei A. Yiakouvaki A. Reelfs O. Santambrogio P. Levi S. Hall N. D. (2014). Role of Intracellular Labile Iron, Ferritin, and Antioxidant Defence in Resistance of Chronically Adapted Jurkat T Cells to Hydrogen Peroxide. Free Radic. Biol. Med. 68, 87–100. 10.1016/j.freeradbiomed.2013.12.006 24333634 PMC4046229

[B2] Amaral E. P. Costa D. L. Namasivayam S. Riteau N. Kamenyeva O. Mittereder L. (2019). A Major Role for Ferroptosis in Mycobacterium Tuberculosis-Induced Cell Death and Tissue Necrosis. J. Exp. Med. 216 (3), 556–570. 10.1084/jem.20181776 30787033 PMC6400546

[B3] Andrews N. C. Schmidt P. J. (2007). Iron Homeostasis. Annu. Rev. Physiol. 69, 69–85. 10.1146/annurev.physiol.69.031905.164337 17014365

[B4] Bersuker K. Hendricks J. M. Li Z. Magtanong L. Ford B. Tang P. H. (2019). The CoQ Oxidoreductase FSP1 Acts Parallel to GPX4 to Inhibit Ferroptosis. Nature 575 (7784), 688–692. 10.1038/s41586-019-1705-2 31634900 PMC6883167

[B5] Borges do Nascimento I. J. Cacic N. Abdulazeem H. M. von Groote T. C. Jayarajah U. Weerasekara I. (2020). Novel Coronavirus Infection (COVID-19) in Humans: A Scoping Review and Meta-Analysis. J. Clin. Med. 9 (4). 10.3390/jcm9040941 PMC7230636 32235486

[B6] Brissot P. Ropert M. Le Lan C. Loréal O. (2012). Non-transferrin Bound Iron: a Key Role in Iron Overload and Iron Toxicity. Biochim. Biophys. Acta (Bba) - Gen. Subjects 1820 (3), 403–410. 10.1016/j.bbagen.2011.07.014 21855608

[B7] Cavezzi A. Troiani E. Corrao S. (2020). COVID-19: Hemoglobin, Iron, and Hypoxia beyond Inflammation. A Narrative Review. Clin. Pract. 10 (2), 1271. 10.4081/cp.2020.1271 32509258 PMC7267810

[B8] Chabot F. Mitchell J. A. Gutteridge J. M. Evans T. W. (1998). Reactive Oxygen Species in Acute Lung Injury. Eur. Respir. J. 11 (3), 745–757. 10.1183/09031936.98.11030745 9596132

[B9] Chen C.-J. Huang H.-S. Chang W.-C. (2003). Depletion of Phospholipid Hydroperoxide Glutathione Peroxidase Up‐regulates Arachidonate Metabolism by 12(S)‐lipoxygenase and Cyclooxygenase 1 in Human Epidermoid Carcinoma A431 Cells. FASEB j. 17 (12), 1694–1696. 10.1096/fj.02-0847fje 12958179

[B10] Chen L. Li X. Liu L. Yu B. Xue Y. Liu Y. (2015). Erastin Sensitizes Glioblastoma Cells to Temozolomide by Restraining xCT and Cystathionine-γ-Lyase Function. Oncol. Rep. 33 (3), 1465–1474. 10.3892/or.2015.3712 25585997

[B11] Chen P. Wu Q. Feng J. Yan L. Sun Y. Liu S. (2020). Erianin, a Novel Dibenzyl Compound in Dendrobium Extract, Inhibits Lung Cancer Cell Growth and Migration via Calcium/calmodulin-dependent Ferroptosis. Sig Transduct Target. Ther. 5 (1), 51. 10.1038/s41392-020-0149-3 PMC7205607 32382060

[B12] Colafrancesco S. Alessandri C. Conti F. Priori R. (2020). COVID-19 Gone Bad: A New Character in the Spectrum of the Hyperferritinemic Syndrome? Autoimmun. Rev. 19 (7), 102573. 10.1016/j.autrev.2020.102573 32387470 PMC7199723

[B13] D'Herde K. Krysko D. V. (2017). Ferroptosis: Oxidized PEs Trigger Death. Nat. Chem. Biol. 13 (1), 4–5. 10.1038/nchembio.2261 27842067

[B14] Dächert J. Schoeneberger H. Rohde K. Fulda S. (2016). RSL3 and Erastin Differentially Regulate Redox Signaling to Promote Smac Mimetic-Induced Cell Death. Oncotarget 7 (39), 63779–63792. 10.18632/oncotarget.11687 27588473 PMC5325403

[B15] Dar H. H. TyurinaMikulska-Ruminska Y. Y. K. Mikulska-Ruminska K. Shrivastava I. Ting H.-C. Tyurin V. A. (2018). *Pseudomonas aeruginosa* Utilizes Host Polyunsaturated Phosphatidylethanolamines to Trigger Theft-Ferroptosis in Bronchial Epithelium. J. Clin. Invest. 128 (10), 4639–4653. 10.1172/jci99490 30198910 PMC6159971

[B16] Deponte M. (2013). Glutathione Catalysis and the Reaction Mechanisms of Glutathione-dependent Enzymes. Biochim. Biophys. Acta (Bba) - Gen. Subjects 1830 (5), 3217–3266. 10.1016/j.bbagen.2012.09.018 23036594

[B17] Dixon S. J. Patel D. N. Welsch M. Skouta R. Lee E. D. Hayano M. (2014). Pharmacological Inhibition of Cystine-Glutamate Exchange Induces Endoplasmic Reticulum Stress and Ferroptosis. eLife 3, e02523. 10.7554/eLife.02523 24844246 PMC4054777

[B18] Dixon S. J. Lemberg K. M. Lamprecht M. R. Skouta R. Zaitsev E. M. Gleason C. E. (2012). Ferroptosis: an Iron-dependent Form of Nonapoptotic Cell Death. Cell 149 (5), 1060–1072. 10.1016/j.cell.2012.03.042 22632970 PMC3367386

[B19] Dixon S. J. Winter G. E. Musavi L. S. Lee E. D. Snijder B. Rebsamen M. (2015). Human Haploid Cell Genetics Reveals Roles for Lipid Metabolism Genes in Nonapoptotic Cell Death. ACS Chem. Biol. 10 (7), 1604–1609. 10.1021/acschembio.5b00245 25965523 PMC4509420

[B20] Doll S. Freitas F. P. Shah R. Aldrovandi M. da Silva M. C. Ingold I. (2019). FSP1 Is a Glutathione-independent Ferroptosis Suppressor. Nature 575 (7784), 693–698. 10.1038/s41586-019-1707-0 31634899

[B21] Dong H. Qiang Z. Chai D. Peng J. Xia Y. Hu R. (2020). Nrf2 Inhibits Ferroptosis and Protects against Acute Lung Injury Due to Intestinal Ischemia Reperfusion via Regulating SLC7A11 and HO-1. Aging 12 (13), 12943–12959. 10.18632/aging.103378 32601262 PMC7377827

[B22] Dowdle W. E. Nyfeler B. Nagel J. Elling R. A. Liu S. Triantafellow E. (2014). Selective VPS34 Inhibitor Blocks Autophagy and Uncovers a Role for NCOA4 in Ferritin Degradation and Iron Homeostasis *In Vivo* . Nat. Cel Biol 16 (11), 1069–1079. 10.1038/ncb3053 25327288

[B23] Edeas M. Saleh J. Peyssonnaux C. (2020). Iron: Innocent Bystander or Vicious Culprit in COVID-19 Pathogenesis? Int. J. Infect. Dis. Official Publ. Int. Soc. Infect. Dis. 97, 303–305. 10.1016/j.ijid.2020.05.110 PMC7264936 32497811

[B24] Frazer D. M. Anderson G. J. (2014). The Regulation of Iron Transport. BioFactors 40 (2), 206–214. 10.1002/biof.1148 24132807

[B25] Frossi B. De Carli M. Piemonte M. Pucillo C. (2008). Oxidative Microenvironment Exerts an Opposite Regulatory Effect on Cytokine Production by Th1 and Th2 Cells. Mol. Immunol. 45 (1), 58–64. 10.1016/j.molimm.2007.05.008 17588662

[B26] Gao M. Monian P. Quadri N. Ramasamy R. Jiang X. (2015). Glutaminolysis and Transferrin Regulate Ferroptosis. Mol. Cel. 59 (2), 298–308. 10.1016/j.molcel.2015.06.011 PMC4506736 26166707

[B27] Ghio A. J. Hilborn E. D. Stonehuerner J. G. Dailey L. A. Carter J. D. Richards J. H. (2008). Particulate Matter in Cigarette Smoke Alters Iron Homeostasis to Produce a Biological Effect. Am. J. Respir. Crit. Care Med. 178 (11), 1130–1138. 10.1164/rccm.200802-334oc 18723436

[B28] Gong Y. Wang N. Liu N. Dong H. (2019). Lipid Peroxidation and GPX4 Inhibition Are Common Causes for Myofibroblast Differentiation and Ferroptosis. DNA Cel. Biol. 38 (7), 725–733. 10.1089/dna.2018.4541 31140862

[B29] Hinman A. Holst C. R. Latham J. C. Bruegger J. J. Ulas G. McCusker K. P. (2018). Vitamin E Hydroquinone Is an Endogenous Regulator of Ferroptosis via Redox Control of 15-lipoxygenase. PloS one 13 (8), e0201369. 10.1371/journal.pone.0201369 30110365 PMC6093661

[B30] Huang C. Wang Y. Li X. Ren L. Zhao J. Hu Y. (2020). Clinical Features of Patients Infected with 2019 Novel Coronavirus in Wuhan, China. The Lancet 395 (10223), 497–506. 10.1016/s0140-6736(20)30183-5 PMC7159299 31986264

[B31] Huang I. Pranata R. Lim M. A. Oehadian A. Alisjahbana B. (2020). C-reactive Protein, Procalcitonin, D-Dimer, and Ferritin in Severe Coronavirus Disease-2019: a Meta-Analysis. Ther. Adv. Respir. Dis. 14, 1753466620937175. 10.1177/1753466620937175 32615866 PMC7336828

[B32] Huang Y. Dai Z. Barbacioru C. Sadée W. (2005). Cystine-glutamate Transporter SLC7A11 in Cancer Chemosensitivity and Chemoresistance. Cancer Res. 65 (16), 7446–7454. 10.1158/0008-5472.can-04-4267 16103098

[B33] Huarte M. (2015). The Emerging Role of lncRNAs in Cancer. Nat. Med. 21 (11), 1253–1261. 10.1038/nm.3981 26540387

[B34] Jaiswal N. N. Bhatnagar H. Shah H. (2020). N-acetycysteine: A Potential Therapeutic Agent in COVID-19 Infection. Med. Hypotheses 144, 110133. 10.1016/j.mehy.2020.110133 32758904 PMC7380211

[B35] Jiang L. Kon N. Li T. Wang S.-J. Su T. Hibshoosh H. (2015). Ferroptosis as a P53-Mediated Activity during Tumour Suppression. Nature 520 (7545), 57–62. 10.1038/nature14344 25799988 PMC4455927

[B36] Jiang N. Zhang X. Gu X. Li X. Shang L. (2021). Progress in Understanding the Role of lncRNA in Programmed Cell Death. Cell Death Discov. 7 (1), 30. 10.1038/s41420-021-00407-1 33558499 PMC7870930

[B37] Kagan V. E. Mao G. Qu F. Angeli J. P. F. Doll S. Croix C. S. (2017). Oxidized Arachidonic and Adrenic PEs Navigate Cells to Ferroptosis. Nat. Chem. Biol. 13 (1), 81–90. 10.1038/nchembio.2238 27842066 PMC5506843

[B38] Kiefmann M. Tank S. Keller P. Börnchen C. Rinnenthal J. L. Tritt M.-O. (2017). IDH3 Mediates Apoptosis of Alveolar Epithelial Cells Type 2 Due to Mitochondrial Ca2+ Uptake during Hypocapnia. Cell Death Dis 8 (8), e3005. 10.1038/cddis.2017.403 28837149 PMC5596584

[B39] Kim K.-H. Maldonado F. Ryu J. H. Eiken P. W. Hartman T. E. Bartholmai B. J. (2010). Iron Deposition and Increased Alveolar Septal Capillary Density in Nonfibrotic Lung Tissue Are Associated with Pulmonary Hypertension in Idiopathic Pulmonary Fibrosis. Respir. Res. 11, 37. 10.1186/1465-9921-11-37 20398288 PMC2867975

[B40] Kim S. Keku T. O. Martin C. Galanko J. Woosley J. T. Schroeder J. C. (2008). Circulating Levels of Inflammatory Cytokines and Risk of Colorectal Adenomas. Cancer Res. 68 (1), 323–328. 10.1158/0008-5472.can-07-2924 18172326 PMC2675825

[B41] Li C. Deng X. Xie X. Liu Y. Friedmann Angeli J. P. Lai L. (2018). Activation of Glutathione Peroxidase 4 as a Novel Anti-inflammatory Strategy. Front. Pharmacol. 9, 1120. 10.3389/fphar.2018.01120 30337875 PMC6178849

[B42] Li D. Wang X. Huang Q. Li S. Zhou Y. Li Z. (2018). Cardioprotection of CAPE-oNO2 against Myocardial Ischemia/reperfusion Induced ROS Generation via Regulating the SIRT1/eNOS/NF-Κb Pathway *In Vivo* and *In Vitro* . Redox Biol. 15, 62–73. 10.1016/j.redox.2017.11.023 29220696 PMC5725281

[B43] Li G. Yang J. Zhao G. Shen Z. Yang K. Tian L. (2021). Dysregulation of Ferroptosis May Involve in the Development of Non‐small‐cell Lung Cancer in Xuanwei Area. J. Cel Mol Med 25 (6), 2872–2884. 10.1111/jcmm.16318 PMC7957160 33528895

[B44] Li J. Lu K. Sun F. Tan S. Zhang X. Sheng W. (2021). Panaxydol Attenuates Ferroptosis against LPS-Induced Acute Lung Injury in Mice by Keap1-Nrf2/HO-1 Pathway. J. Transl Med. 19 (1), 96. 10.1186/s12967-021-02745-1 33653364 PMC7927246

[B45] Li X. Duan L. Yuan S. Zhuang X. Qiao T. He J. (2019). Ferroptosis Inhibitor Alleviates Radiation-Induced Lung Fibrosis (RILF) via Down-Regulation of TGF-Β1. J. Inflamm. 16, 11. 10.1186/s12950-019-0216-0 PMC6542066 31160885

[B46] Li Y. Cao Y. Xiao J. Shang J. Tan Q. Ping F. (2020). Inhibitor of Apoptosis-Stimulating Protein of P53 Inhibits Ferroptosis and Alleviates Intestinal Ischemia/reperfusion-Induced Acute Lung Injury. Cell Death Differ 27 (9), 2635–2650. 10.1038/s41418-020-0528-x 32203170 PMC7429834

[B47] Linkermann A. Stockwell B. R. Krautwald S. Anders H.-J. (2014). Regulated Cell Death and Inflammation: an Auto-Amplification Loop Causes Organ Failure. Nat. Rev. Immunol. 14 (11), 759–767. 10.1038/nri3743 25324125

[B48] Liu P. Feng Y. Li H. Chen X. Wang G. Xu S. (2020). Ferrostatin-1 Alleviates Lipopolysaccharide-Induced Acute Lung Injury via Inhibiting Ferroptosis. Cell Mol Biol Lett 25, 10. 10.1186/s11658-020-00205-0 32161620 PMC7045739

[B49] Liu W. Zhang S. Nekhai S. Liu S. (2020). Depriving Iron Supply to the Virus Represents a Promising Adjuvant Therapeutic against Viral Survival. Curr. Clin. Microbiol. Rep. 20, 1–7. 10.1007/s40588-020-00140-w PMC7169647 32318324

[B50] Ma Q. (2013). Role of Nrf2 in Oxidative Stress and Toxicity. Annu. Rev. Pharmacol. Toxicol. 53, 401–426. 10.1146/annurev-pharmtox-011112-140320 23294312 PMC4680839

[B51] Mak T. W. Grusdat M. Duncan G. S. Dostert C. Nonnenmacher Y. Cox M. (2017). Glutathione Primes T Cell Metabolism for Inflammation. Immunity 46 (4), 675–689. 10.1016/j.immuni.2017.03.019 28423341

[B52] Mao C. Wang X. Liu Y. Wang M. Yan B. Jiang Y. (2018). A G3BP1-Interacting lncRNA Promotes Ferroptosis and Apoptosis in Cancer via Nuclear Sequestration of P53. Cancer Res. 78 (13), 3484–3496. 10.1158/0008-5472.CAN-17-3454 29588351 PMC8073197

[B53] Matsushita M. Freigang S. Schneider C. Conrad M. Bornkamm G. W. Kopf M. (2015). T Cell Lipid Peroxidation Induces Ferroptosis and Prevents Immunity to Infection. J. Exp. Med. 212 (4), 555–568. 10.1084/jem.20140857 25824823 PMC4387287

[B54] McLaughlin K. M. Bechtel M. Bojkova D. Münch C. Ciesek S. Wass M. N. (2020). COVID-19-Related Coagulopathy-Is Transferrin a Missing Link? Diagnostics (Basel) 10 (8), 539. 10.3390/diagnostics10080539 PMC7459734 32751741

[B55] Muhoberac B. B. (2020). What Can Cellular Redox, Iron, and Reactive Oxygen Species Suggest about the Mechanisms and Potential Therapy of COVID-19? Front. Cel. Infect. Microbiol. 10, 569709. 10.3389/fcimb.2020.569709 PMC7767833 33381464

[B56] Murphy T. H. Miyamoto M. Sastre A. Schnaar R. L. Coyle J. T. (1989). Glutamate Toxicity in a Neuronal Cell Line Involves Inhibition of Cystine Transport Leading to Oxidative Stress. Neuron 2 (6), 1547–1558. 10.1016/0896-6273(89)90043-3 2576375

[B57] Ou Y. Wang S.-J. Li D. Chu B. Gu W. (2016). Activation of SAT1 Engages Polyamine Metabolism with P53-Mediated Ferroptotic Responses. Proc. Natl. Acad. Sci. USA 113 (44), E6806–E6812. 10.1073/pnas.1607152113 27698118 PMC5098629

[B58] Pan Y. Tang P. Cao J. Song Q. Zhu L. Ma S. (2020). Lipid Peroxidation Aggravates Anti-tuberculosis Drug-Induced Liver Injury: Evidence of Ferroptosis Induction. Biochem. biophysical Res. Commun. 533 (4), 1512–1518. 10.1016/j.bbrc.2020.09.140 33121683

[B59] Park E.-J. Park Y.-J. Lee S. J. Lee K. Yoon C. (2019). Whole Cigarette Smoke Condensates Induce Ferroptosis in Human Bronchial Epithelial Cells. Toxicol. Lett. 303, 55–66. 10.1016/j.toxlet.2018.12.007 30579903

[B60] Persson H. L. Nilsson K. J. Brunk U. T. (2001). Novel Cellular Defenses against Iron and Oxidation: Ferritin and Autophagocytosis Preserve Lysosomal Stability in Airway Epithelium. Redox Rep. 6 (1), 57–63. 10.1179/135100001101536049 11333118

[B61] Persson H. L. Vainikka L. K. Eriksson H. B. Wennerström U. (2011). Lane-Hamilton Syndrome. Chest 139 (2), 361–367. 10.1378/chest.10-0818 20705801

[B62] Pi J. Zhang Q. Woods C. G. Wong V. Collins S. Andersen M. E. (2008). Activation of Nrf2-Mediated Oxidative Stress Response in Macrophages by Hypochlorous Acid. Toxicol. Appl. Pharmacol. 226 (3), 236–243. 10.1016/j.taap.2007.09.016 17980396

[B63] Proneth B. Conrad M. (2019). Ferroptosis and Necroinflammation, a yet Poorly Explored Link. Cel Death Differ 26 (1), 14–24. 10.1038/s41418-018-0173-9 PMC6294786 30082768

[B64] Qiang Z. Dong H. Xia Y. Chai D. Hu R. Jiang H. (2020). Nrf2 and STAT3 Alleviates Ferroptosis-Mediated IIR-ALI by Regulating SLC7A11. Oxidative Med. Cell. longevity 2020, 5146982. 10.1155/2020/5146982 PMC7520693 33014271

[B65] Rahman I. Skwarska E. Henry M. Davis M. O'Connor C. M. FitzGerald M. X. (1999). Systemic and Pulmonary Oxidative Stress in Idiopathic Pulmonary Fibrosis. Free Radic. Biol. Med. 27 (1-2), 60–68. 10.1016/s0891-5849(99)00035-0 10443920

[B66] Reuter S. Gupta S. C. Chaturvedi M. M. Aggarwal B. B. (2010). Oxidative Stress, Inflammation, and Cancer: How Are They Linked? Free Radic. Biol. Med. 49 (11), 1603–1616. 10.1016/j.freeradbiomed.2010.09.006 20840865 PMC2990475

[B67] Richeldi L. Collard H. R. Jones M. G. (2017). Idiopathic Pulmonary Fibrosis. The Lancet 389 (10082), 1941–1952. 10.1016/s0140-6736(17)30866-8 28365056

[B68] Roh J.-L. Kim E. H. Jang H. Shin D. (2017). Nrf2 Inhibition Reverses the Resistance of Cisplatin-Resistant Head and Neck Cancer Cells to Artesunate-Induced Ferroptosis. Redox Biol. 11, 254–262. 10.1016/j.redox.2016.12.010 28012440 PMC5198738

[B69] Seyedrezazadeh E. Ostadrahimi A. Mahboob S. Assadi Y. Ghaemmagami J. Pourmogaddam M. (2008). Effect of Vitamin E and Selenium Supplementation on Oxidative Stress Status in Pulmonary Tuberculosis Patients. Respirology 13 (2), 294–298. 10.1111/j.1440-1843.2007.01200.x 18339032

[B70] Shimada K. Hayano M. Pagano N. C. Stockwell B. R. (2016). Cell-Line Selectivity Improves the Predictive Power of Pharmacogenomic Analyses and Helps Identify NADPH as Biomarker for Ferroptosis Sensitivity. Cel Chem. Biol. 23 (2), 225–235. 10.1016/j.chembiol.2015.11.016 PMC4792701 26853626

[B71] Shin D. Kim E. H. Lee J. Roh J.-L. (2018). Nrf2 Inhibition Reverses Resistance to GPX4 Inhibitor-Induced Ferroptosis in Head and Neck Cancer. Free Radic. Biol. Med. 129, 454–462. 10.1016/j.freeradbiomed.2018.10.426 30339884

[B72] Silvagno F. Vernone A. Pescarmona G. P. (2020). The Role of Glutathione in Protecting against the Severe Inflammatory Response Triggered by COVID-19. Antioxidants (Basel) 9 (7). 10.3390/antiox9070624 PMC7402141 32708578

[B73] Stoyanovsky D. A. Tyurina Y. Y. Shrivastava I. Bahar I. Tyurin V. A. Protchenko O. (2019). Iron Catalysis of Lipid Peroxidation in Ferroptosis: Regulated Enzymatic or Random Free Radical Reaction? Free Radic. Biol. Med. 133, 153–161. 10.1016/j.freeradbiomed.2018.09.008 30217775 PMC6555767

[B74] Sun X. Ou Z. Chen R. Niu X. Chen D. Kang R. (2016). Activation of the P62-Keap1-NRF2 Pathway Protects against Ferroptosis in Hepatocellular Carcinoma Cells. Hepatology 63 (1), 173–184. 10.1002/hep.28251 26403645 PMC4688087

[B75] Suzuki T. Motohashi H. Yamamoto M. (2013). Toward Clinical Application of the Keap1-Nrf2 Pathway. Trends Pharmacological Sciences 34 (6), 340–346. 10.1016/j.tips.2013.04.005 23664668

[B76] Tang D. Chen X. Kang R. Kroemer G. (2021). Ferroptosis: Molecular Mechanisms and Health Implications. Cell Res 31 (2), 107–125. 10.1038/s41422-020-00441-1 33268902 PMC8026611

[B77] Wan C. Li S. Wen L. Kong J. Wang K. Zhu Y. (2007). Damage of Oxidative Stress on Mitochondria during Microspores Development in Honglian CMS Line of rice. Plant Cel Rep 26 (3), 373–382. 10.1007/s00299-006-0234-2 17053903

[B78] Wang M. Mao C. Ouyang L. Liu Y. Lai W. Liu N. (2019). Long Noncoding RNA LINC00336 Inhibits Ferroptosis in Lung Cancer by Functioning as a Competing Endogenous RNA. Cel Death Differ 26 (11), 2329–2343. 10.1038/s41418-019-0304-y PMC6889193 30787392

[B79] Wang S.-J. Li D. Ou Y. Jiang L. Chen Y. Zhao Y. (2016). Acetylation Is Crucial for P53-Mediated Ferroptosis and Tumor Suppression. Cel Rep. 17 (2), 366–373. 10.1016/j.celrep.2016.09.022 PMC5227654 27705786

[B80] Wang Y. Tang M. (2019). PM2.5 Induces Ferroptosis in Human Endothelial Cells through Iron Overload and Redox Imbalance. Environ. Pollut. (Barking, Essex :1987) 254 (Pt A), 112937. 10.1016/j.envpol.2019.07.105 31401526

[B81] Wen Q. Liu J. Kang R. Zhou B. Tang D. (2019). The Release and Activity of HMGB1 in Ferroptosis. Biochem. biophysical Res. Commun. 510 (2), 278–283. 10.1016/j.bbrc.2019.01.090 30686534

[B82] Wu Y. Chen H. N. Xuan. Xuan N. Zhou L. Wu Y. Zhu C. (2020). Induction of Ferroptosis-like Cell Death of Eosinophils Exerts Synergistic Effects with Glucocorticoids in Allergic Airway Inflammation. Thorax 75 (11), 918–927. 10.1136/thoraxjnl-2020-214764 32759385

[B83] Xia F. Chen H. Jin Z. Fu Z. (2021). Apelin-13 Protects the Lungs from Ischemia-Reperfusion Injury by Attenuating Inflammatory and Oxidative Stress. Hum. Exp. Toxicol. 40 (4), 685–694. 10.1177/0960327120961436 33025833

[B84] Xie Y. Hou W. Song X. Yu Y. Huang J. Sun X. (2016). Ferroptosis: Process and Function. Cel Death Differ 23 (3), 369–379. 10.1038/cdd.2015.158 PMC5072448 26794443

[B85] Xu Y. Li X. Cheng Y. Yang M. Wang R. (2020). Inhibition of ACSL4 Attenuates Ferroptotic Damage after Pulmonary Ischemia‐reperfusion. FASEB j. 34 (12), 16262–16275. 10.1096/fj.202001758r 33070393

[B86] Yang W. S. Kim K. J. Gaschler M. M. Patel M. Shchepinov M. S. Stockwell B. R. (2016). Peroxidation of Polyunsaturated Fatty Acids by Lipoxygenases Drives Ferroptosis. Proc. Natl. Acad. Sci. USA 113 (34), E4966–E4975. 10.1073/pnas.1603244113 27506793 PMC5003261

[B87] Yokoi E. Mabuchi S. Komura N. Shimura K. Kuroda H. Kozasa K. (2019). The Role of Myeloid-Derived Suppressor Cells in Endometrial Cancer Displaying Systemic Inflammatory Response: Clinical and Preclinical Investigations. Oncoimmunology 8 (12), e1662708. 10.1080/2162402x.2019.1662708 31741758 PMC6844305

[B88] Yoshida M. Minagawa S. Araya J. Sakamoto T. Hara H. Tsubouchi K. (2019). Involvement of Cigarette Smoke-Induced Epithelial Cell Ferroptosis in COPD Pathogenesis. Nat. Commun. 10 (1), 3145. 10.1038/s41467-019-10991-7 31316058 PMC6637122

[B89] Zhang Y. Zhuang L. Gan B. (2019). BAP1 Suppresses Tumor Development by Inducing Ferroptosis upon SLC7A11 Repression. Mol. Cell Oncol. 6 (1), 1536845. 10.1080/23723556.2018.1536845 30788415 PMC6370386

[B90] Zhao J. Dar H. H. Deng Y. St Croix C. M. Li Z. Minami Y. (2020). PEBP1 Acts as a Rheostat between Prosurvival Autophagy and Ferroptotic Death in Asthmatic Epithelial Cells. Proc. Natl. Acad. Sci. U S A. 117 (25), 14376–14385. 10.1073/pnas.1921618117 32513718 PMC7321965

[B91] Zhao J. O'Donnell V. B. Balzar S. St. Croix C. M. Trudeau J. B. Wenzel S. E. (2011). 15-Lipoxygenase 1 Interacts with Phosphatidylethanolamine-Binding Protein to Regulate MAPK Signaling in Human Airway Epithelial Cells. Proc. Natl. Acad. Sci. 108 (34), 14246–14251. 10.1073/pnas.1018075108 21831839 PMC3161579

[B92] Zhao K. Huang J. Dai D. Feng Y. Liu L. Nie S. (2020). Serum Iron Level as a Potential Predictor of Coronavirus Disease 2019 Severity and Mortality: A Retrospective Study. Open Forum Infect. Dis. 7 (7), ofaa250. 10.1093/ofid/ofaa250 32661499 PMC7337740

[B93] Zhou F. Yu T. Du R. Fan G. Liu Y. Liu Z. (2020). Clinical Course and Risk Factors for Mortality of Adult Inpatients with COVID-19 in Wuhan, China: a Retrospective Cohort Study. The Lancet 395 (10229), 1054–1062. 10.1016/s0140-6736(20)30566-3 PMC7270627 32171076

[B94] Zhou H. Li F. Niu J. Y. Zhong W. Y. Tang M. Y. Lin D. (2019). Ferroptosis Was Involved in the Oleic Acid-Induced Acute Lung Injury in Mice. Sheng Li Xue Bao 71 (5), 689–697. 10.13294/j.aps.2019.0070 31646322

[B95] Zhou Y. Que K.-T. Zhang Z. Yi Z. J. Zhao P. X. You Y. (2018). Iron Overloaded Polarizes Macrophage to Proinflammation Phenotype through ROS/acetyl-p53 Pathway. Cancer Med. 7 (8), 4012–4022. 10.1002/cam4.1670 29989329 PMC6089144

